# Authors' Response to Peer Reviews of “COVID-19 Outcomes and Genomic Characterization of SARS-CoV-2 Isolated From Veterans in New England States: Retrospective Analysis”

**DOI:** 10.2196/35515

**Published:** 2021-12-17

**Authors:** Megan Lee, Ya Haddy Sallah, Mary Petrone, Matthew Ringer, Danielle Cosentino, Chantal B F Vogels, Joseph R Fauver, Tara D Alpert, Nathan D Grubaugh, Shaili Gupta

**Affiliations:** 1 Yale School of Medicine West Haven, CT United States; 2 Yale School of Public Health New Haven, CT United States; 3 VA Connecticut Healthcare System West Haven, CT United States

**Keywords:** infectious disease, COVID-19, epidemiology, veteran, outcome, sequencing, genetics, virus, United States, impact, testing, severity, mortality, cohort

*This is the authors’ response to peer-review reports for “COVID-19 Outcomes and Genomic Characterization of SARS-CoV-2 Isolated From Veterans in New England States: Retrospective Analysis.”*

## Round 1 Review

### Reviewer W [1]

#### General Comments

The sudden menace imposed by the COVID-19 pandemic has led to the proliferation of studies on the epidemiology of viral genomics, specifically to understand disease risk factors, characteristics, and prognosis of those with COVID-19 [2-4]. Between 20% to 40% of COVID-19 admissions are reported to require intensive care [5], and have a fatality rate of 35% to 50% [6]. Many factors have been reported to either account for or to be associated with the clinical characteristics and prognosis of patients with COVID-19 [7-9]. Given that the aforementioned body of knowledge among veterans in New England is currently limited, the authors of the paper titled “COVID-19 Outcomes and Genomic Characterization of SARS-CoV-2 Isolated From Veterans in New England States” [10] investigated the patient characteristics, comorbidities, and disease predictors in a cohort of 426 veterans hospitalized for COVID-19. They found using a multivariate regression that age was the most significant predictor of being hospitalized, the severity of disease, and mortality; being non-White was more associated with being hospitalized; and those in need of oxygen upon admission were more likely to die.

Even though widely reported, genomic epidemiology remains a rapidly growing domain in virology [11]. Besides, the diversity of the four coronavirus genera (alpha, beta, gamma, and delta) [12] and the emergence and spreading of the B.1.1.7 variant from the United Kingdom, B.1.1.28 from Brazil, and B.1.351 from South Africa [13] warrant constant new data and knowledge translation. To this effect, this paper addresses a major area of concern and interest to the readership of the journal. The authors are clear in their title, which still needs to fully comply with the journal guidelines. The Abstract follows the guidelines and presents an overview of the study. Being an area that has received tremendous interest since the start of the COVID-19 pandemic, there was an overriding need for this study to be put in context. The paper’s introduction does well, ends with the study aim, and is brief at highlighting the main concern but deserves more attention. The general structure of the paper needs improvement to comply with the journal guidelines. The data collection methods, albeit needing clarification, seem reasonable with appropriate analysis, thereby giving value to the results. The discussion of the paper has been well articulated, and the conclusion ties with the research objective. The English used is simple and in plain language for easy comprehension.

Although congratulating the authors for a good attempt and concise paper, the paper will benefit from more value if the following specific comments are given consideration.

Response: We thank the reviewer for the careful review of this paper and the summary. Their feedback has certainly improved this manuscript. Our response to each comment is in the following sections.

#### Specific Comments

1. The general structure of the paper needs to conform to the journal guidelines.

Response: We have reformatted the paper to conform to journal guidelines.

2. The paper deserves to be put in context to be more appealing.

Response: We have added more context throughout the paper to achieve this. The main areas where this is reflected now are in the Study Rationale subsection (Introduction) and throughout the Results and Discussion sections.

3. The introduction appears too restrictive and could be made more robust.

Response: We thank the reviewer for this suggestion. This has been done now.

4. The methods and reported results warrant the use of appropriate guidelines.

Response: This has been done now.

5. All tables and figures need to be formatted following the guidelines.

Response: This has been done now.

6. Your references need slight improvement, in line with the guidelines.

Response: We have reformatted based on journal guidelines and have also added relevant references based on reviewer suggestions.

To elucidate the aforementioned specific comments, kindly refer to the major and minor comments.

##### Major Comments

1. Kindly format your title following the guidelines [14]. Your title should normally end with a study design after a semicolon.

Response: We have edited our title to be: “COVID-19 Outcomes and Genomic Characterization of SARS-CoV-2 Isolated From Veterans in New England States: A Retrospective Analysis.”

2. The methods subsection of the Abstract needs to summarize the study design; total sample, setting, and recruitment; mean age and gender differences; end points measured; data collection procedure; and data analysis. You may want to change the subtitle from “Study Design” to “Methods.”

Response: Thank you. We have modified the Abstract to provide age and gender differences in the target population as well as the actual subject population. Subsections have been added to the Abstract as suggested.

3. Kindly use the following template to give your paper an overall structure that complies with the journal guidelines [15].

Response: We have formatted our manuscript according to the template provided.

4. Given the high amount of reported literature in this field, I suggest putting your study in context [2]. Kindly search the Cochrane and Pubmed databases to:

1) Summarize the evidence already reported on the topic

2) Report why this study was necessary and the value added to the existing literature

3) The implication of all available evidence (including that from this study)

Response: We thank the reviewer for this comment. It has certainly made our Introduction section stronger. We have added references and language on the implication and relevance of evidence in this area of work in the field of COVID-19. Please see our full introduction for the additions and modifications, pages 2-3, lines 60-98.

5. It will be good to structure your Introduction into Background, Study Rationale, and Study Aim.

Response: We have added these subheadings to our Introduction section.

6. Kindly structure your Methods section and report it as follows:

1) Specific objectives

2) Study design with justification (kindly make clear if this was a retrospective or prospective cohort study)

3) Study setting

4) Sample size calculations

5) Participant recruitment (with inclusion and exclusion criteria)

6) Sample/data collection

7) Sample handling procedure and quality control

8) Outcome measures (indicate whether these were continuous, binary, or categorical).

9) Whole genome sequencing (WGS) and phylogenetic analysis

10) Data analysis (with justification for the approach used)

11) Ethical considerations

Response: We have structured the Methods section with all these subheadings. Specific comments about methods will be addressed in the following points.

7. It is not clear whether this was a retrospective study since patients were still hospitalized at the time of this study. In 6.2 above, kindly be precise about the type of cohort study you undertook.

Response: This was a retrospective study, which we have now stated clearly in our Methods section, page 6, line 106: “We conducted a retrospective chart review to gather the demographic and clinical variables.”

For further clarification of our methods, we included the following in our Methods section, page 7, lines 131-134: “All data collection was retrospective after a diagnosis of COVID-19 had been confirmed. If chart review occurred while a veteran was hospitalized, the chart was again reviewed retrospectively after discharge from hospital.”

8. As part of your participant recruitment, indicate attempts made to reduce bias.

Response: This was a retrospective study, so we did not recruit participants. As indicated in our Methods section, we included all veterans who were diagnosed with COVID-19 in this era with accessible medical records.

9. In 6.6 above, give details of those that collected samples and how that was done. If this was done by your research team, ensure to report the protocol used to collect samples. Organize your data collection into:

1) Hospitalization data

2) Peak disease severity data

3) Mortality data

4) Genome sequencing data

Response: This has been defined clearly now in the Methods section and the subsections Data Collection, Sample Collection, Whole Genome Sequencing, and Outcome Measures.

10. In 6.7 above, kindly clarify how samples were handled (including storage). If this was not done by the research team and was only reported, kindly indicate as such. If samples were not collected by you, provide details on how you had access to samples.

Response: This has been clarified now in the subsection Sample Collection and Handling, page 7, lines 136-144: “Sample collection and handling: Handling of nasopharyngeal specimens or isolated virus was carried out by the VACHS clinical laboratory as part of clinical care, following standardized CLIA guidelines. Our viral repository was populated by the positive test results of all New England veterans. VACHS laboratory handled specimens, isolated the SARS-CoV-2 RNA, and shipped it for whole genome sequencing (WGS) to non-VA laboratory. We obtained the details of platform used to diagnose, the cycle threshold, and the date of test from the laboratory. Sequencing of viral genome was conducted at the non-VA laboratory by our co-authors as follows.”

11. In 6.9 above, it is important to report the protocol/guidelines you used in genome sequencing. You may want to justify your procedure using these WHO guidelines [16] as well as substantiating your procedure with a visual display/flow of how the sequencing works.

Response: We thank the reviewer for this comment and would like to provide clarification. The genome sequencing method and the alignment approach are defined clearly in the subsection on WGS. Assignment of lineages was with Pangolin as described. Citations have been provided for reference. Any further granular detail on this method would be out of the scope of this paper.

12. As part of your statistical analysis, could you please justify your use of nonparametric tests? Kindly report the normality tests that were performed and the figures.

Response: We used logistic regressions to model the outcomes of hospitalization and mortality, and ordinal logistic regression to model peak disease severity because the outcomes were categorical and ordinal, respectively. Logistic and original logistic regressions do not require an assumption of normality. We have edited our paper to make this clearer, page 8, lines 168 and 169: “We used STATA v16 (College Station, TX) for logistic regressions to predict our hospitalization and mortality, and ordinal logistic regression to predict peak disease severity.”

13. It might be worth arranging your data analysis first into univariate analysis and multivariate analysis, and then into hospitalization, peak disease severity, mortality, and genome sequencing.

Response: We have rephrased our Methods section to make the structure of analysis more clear, pages 8 and 9, lines 168-172: “We used STATA v16 (College Station, TX) for logistic regressions to predict our hospitalization and mortality, and ordinal logistic regression to predict peak disease severity. We first conducted a univariate analysis, then used significant variables from the univariate analysis (*P*< 0.05) to use in a multivariate model for each of our outcomes to assess the impact of several variables at once, which has been frequently used in COVID-19 literature. Genomic characteristics were reported descriptively.”

14. In your data analysis, kindly report how you moved from univariate to multivariate analysis or how you selected variables for your multivariate model.

Response: We agree with the reviewer that more clarification is necessary, so we have described our methods in more detail, Page 8 and 9, lines 169-172: “We first conducted a univariate analysis, then used significant variables from the univariate analysis (*P*< 0.05) to use in a multivariate model for each of our outcomes to assess the impact of several variables at once, which has been frequently used in COVID-19 literature.”

15. It is very important to indicate the guidelines used to report your review results. As part of your ethical considerations, indicate the guidelines you used to report your results. You may want to use these depending on which best suits your study method [17,18].

Response: We thank the reviewer. We have cited the Record statement for this. Our report follows those guidelines, page 9, lines 181 and 182: “RECORD statement guidelines were used to maintain transparency in the reporting of this work.”

16. Your Results section should be reported in line with the Methods section starting with the participant characteristics. You might want to report your results as follows:

1) Participant characteristics

2) Predictors of hospitalization

3) Predictors of peak disease severity

4) Predictors of mortality

5) Genome sequencing and phylogenetics

Response: We thank the reviewer for the comments, and we have organized the Results section into three headings to make it more clear for the reader: (1) Participant Characteristics; (2) Rates and Predictors of Hospitalization, Peak Severity, and Mortality; and (3) Genomic Characteristics

17. Kindly move your Supplemental Table 1 to Participant Characteristics in the Results section.

Response: We have moved Supplemental Table 1 to the Results section on page 10 and have renamed it Table 1.

18. Kindly move Supplemental Figure 1 and Supplemental Figure 2 to the Predictors of Hospitalization and Predictors of Mortality subsections of the Results section, respectively.

Response: We have moved Supplemental Figures 1 and 2 to the Results section on pages 13 and 14, and renamed them Figure 1 and Figure 2.

19. Note that the whole of your manuscript must be in portrait. You may want to highlight your Table 1 then click on “fit to window” on the automatic adjustment tab of Microsoft Word and move it together with Figure 1 to the Genomic Sequencing subsection of your Results section.

Response: We thank the reviewer for this comment, and we have adjusted Table 1 so that it fits within a portrait page.

20. In the presentation of the results of your logistic regression, it will be good to state how the following assumptions were met:

1) Binary outcome

2) Linearity

3) Outliers

4) Multicollinearity

Response: We thank the reviewer for the comment and have included the following sentence in the Methods section, page 9, lines 173 and 174: “Assumptions for logistic regressions (binary outcome, linearity, no outliers, and multicollinearity) were tested and met, with maximum variance inflation factors of 2.”

21. As part of the reported results of your regression, I suggest proving an explanation on your model’s goodness of fit by plotting and reporting the area under the receiver operating characteristic (ROC) curve.

Response: We agree with the reviewer, and we have provided the area under the ROC curve (the C-statistic) for our multivariate models in the text of the Results section, page 11, lines 207-213: “In multivariate regression, significant predictors of hospitalization (C-statistic: 0.75) were age (OR: 1.05, 95% CI: 1.03, 1.08) and non-White race (OR: 2.39, 95% CI: 1.13, 5.01) (Table 3). Peak severity (C-statistic: 0.70) also varied by age (OR: 1.07, 95% CI: 1.03, 1.11) and O2 requirement on admission (OR: 45.7, 95% CI: 18.79, 111). Mortality (C-statistic: 0.87) was predicted by age (OR: 1.06, 1.01, 1.11), dementia (OR:3.44, 95% CI: 1.07, 11.1), and O2 requirement on admission (OR: 6.74, 95% CI: 1.74, 26.1).”

22. Kindly follow the guidelines to structure your Discussion section as follows:

1) Principal findings (summary)

2) Comparison with prior studies

3) Study limitations

Response: We have structured the Discussion section in this format and have added subheadings with the exact wording.

23. Include a subsection “Author Contribution” after the Acknowledgments section to state the contribution of each author included in this paper.

Response: We have included author contributions on page 19, lines 348-354: “Author contributions: The authors confirm contribution to the manuscript as follows: ML and SG participated in the conception, design, data collection, analysis and interpretation of results, and manuscript preparation. YHS and MR participated in the data collection, analysis and interpretation of results, and manuscript preparation. MEP and NDG participated in the conduction, analysis and interpretation of whole genome sequencing, and in manuscript preparation. DC participated in the data collection, analysis and interpretation of results. CBFV, JRF and TA participated in the conduction and analysis of whole genome sequencing.”

24. Include a subsection “Conflicts of Interest” after “Author Contributions” to declare any conflict of interest.

Response: We have included the following conflict of interest on Page 19, Line 360: “Conflict of interest: NDG is a paid consultant of Tempus Labs for infectious disease genomics..”

25. Kindly list all Multimedia Appendices before the References section. For instance, your supplemental Table 2 will be labeled in the body of the manuscript as follows:

- Multimedia Appendix 1: Genomic lineage

Response: We have labeled all multimedia appendices before the References section.

26. Create a section “Abbreviations” after your references to list and expand all abbreviations in the text.

Response: We have created an “Abbreviations” page after our references to list and expand all abbreviations in the text, page 26:

“BMI: body mass index

CAD: coronary artery disease

CKD: chronic kidney disease

COPD: chronic obstructive pulmonary disease

COVID-19: coronavirus disease of 2019

IRB: Institutional Review Board

L: Liters

LTC: long term care

SARS-CoV-2: severe acute respiratory syndrome coronavirus 2

O2: oxygen

OR: odds ratio

OSA: obstructive sleep apnea

VA: Veterans Administration

VACHS: Veterans Administration Connecticut”

##### Minor Comments

27. You may want to include just the corresponding author on the manuscript and add all other authors in the metadata section of the online manuscript management system.

Response: Because we had enough space on the manuscript title page and for stylistic reasons, we have chosen to include all authors on the title page.

28. Kindly format your tables following the journal guidelines [19].

Response: We have formatted the tables according to guidelines.

29. Kindly number your tables in the body of the text in order of appearance (Table 1, 2, 3, etc).

Response: We have renumbered all the tables in order of appearance in the manuscript.

30. You need to report any *P* values based on the guidelines (eg, *P*=.05 or *P*<.001).

Response: We have reported all calculated *P* values in our manuscript according to the journal’s guidelines.

31. Review all your figures and their captions to ensure they are in line with the guidelines [20]. Apart from being uploaded as multimedia appendices, all figures must appear in the body of the text where they are first mentioned. The caption of each figure must appear at the bottom of the figure.

Response: We have moved all tables and figures up to where they should be in the text and added captions below each figure.

32. In your Discussion section, it will be appropriate to organize the “Comparison With Prior Studies” into subtitles as follows:

1) Predictors of hospitalization

2) Predictors of peak disease severity

3) Predictors of mortality

4) Genomic sequencing

Response: We thank the reviewer for this comment. We considered this but found that dividing the first part of the discussion into these four subheadings would result in small subsections. We instead took the reviewer’s prior suggestion of dividing the discussion into three subsections: Principal Findings, Comparison With Prior Studies, and Limitations. Our Discussion section has been strengthened by this.

33. I suggest starting your conclusion with a statement on the study objectives followed by a summary of findings, then lessons learned from your findings, and finally suggested direction of future research.

Response: We thank the reviewer for the suggestion and have reframed the first paragraph of our introduction to fit with the reviewer’s suggestions, pages 15 and 16, lines 288-300: “Our study found that in a cohort of veterans with average age of 63 years and a high comorbidity burden, age significantly associated with risk of hospitalization, peak disease severity, and mortality. O2 requirement upon admission correlated with peak disease severity and mortality, while dementia was an additional factor associated with higher mortality. The CDC provides a list of chronic medical conditions (May 2021) that predispose individuals to severe illness from SARS-CoV-2 infection [21], but >75% of United States adults fall under a high-risk category [22]. Veterans are a unique cohort because of advanced age on average [23], and more comorbidities. Understanding clinical factors that impact outcomes in veterans will help healthcare providers risk-stratify patients with similar demographic profiles, and future research should explore the impact of new treatments and vaccination on outcomes. The predominance of B lineage D614G in our study specimens provided valuable insight into the pace of epidemiological trend and evolution of the virus early in the COVID-19 era through the New England region.”

34. You need to delete your “Supplemental Table 2. Lineages of genomes” from the manuscript and upload it as a Multimedia Appendix in the online manuscript submission system. All multimedia appendices must be referenced in the body of your paper. Kindly have a look at other papers published in JMIRx Med.

Response: We have changed this to “Multimedia Appendix 1,” as previously mentioned in point 25.

35. Kindly make Acknowledgments, Funding, and Conflicts of Interest subsections.

Response: We have made each of these sections as subsections, along with “Author Contributions.”

36. Your references need to be formatted following the journal guidelines. Set your reference manager to the American Medical Association (AMA) citation style and make sure to include a PubMed ID at the end of each reference. You can search the PubMed IDs of articles at https://pubmed.ncbi.nlm.nih.gov/. It is also possible to copy your citation directly from the PubMed site provided it has been set to the AMA style (see references to this report for examples).

For articles without PMIDs, kindly include a DOI and ensure you verify your DOIs using https://www.doi.org/ to make sure they work.

Response: We have edited our references to include PMIDs whenever available and formatted them according to journal guidelines.

37. For referenced websites, ensure to make as much effort as possible to get and reference the PDF version of the article (ie, in the absence of a PMID and DOI).

Response: We have made every effort to reference PDF versions of articles whenever possible.

### Reviewer AV [24]

#### General Comments

The authors presented a study about the clinical and genomic characterization of COVID-19 from a veteran group. I have some questions for the authors.

1. Line 85: Authors wrote, “we recorded hospitalization status, mortality, and oxygen (O2)-requirement within 24 hours of admission.” Here, can authors clarify if they recorded each single patient’s clinical information within 24 hours of admission or they collected them from chart review? In addition, for O2, the 2 should be subscript.

Response: We thank the reviewer for helping us clarify this. We did gather this information from manual chart review and have updated our methods to read, page 8, lines 160 and 161: “Our categorical outcomes, also derived from manual chart review, were hospitalization status, mortality, and oxygen (O2)-requirement within 24 hours of admission from manual chart review.”

We have also changed O2 throughout the manuscript to have a subscript.

2. Lines 105 and 106: The disease name should be capitalized.

Response: We thank the reviewer for this comment; however, disease names are not typically capitalized unless they are an abbreviation.

3. Line 113: Authors did not provide a transition between the univariate regression and multivariate regression. Univariate analysis was simply mentioned in the first sentence without any explanation or discussion. Authors should indicate the reason why they conducted multivariate analysis (eg, univariate was not specific enough). Additionally, in general, the factors should have the first letter capitalized, for example, Age, non-White race.

Response: We thank the reviewer for this comment. As in our response to reviewer W, we have edited our description and clarified our univariate and multivariate analyses, pages 8 and 9, lines 168-172: “We used STATA v16 (College Station, TX) for logistic regressions to predict our hospitalization and mortality, and ordinal logistic regression to predict peak disease severity. We first conducted a univariate analysis, then used significant variables from the univariate analysis (*P* < 0.05) to use in a multivariate model for each of our outcomes to assess the impact of several variables at once, which has been frequently used in COVID-19 literature.”

We have ensured that White and non-White are capitalized where present. Age is usually not capitalized.

4. Line 129: Authors wrote, “our study found that in an older cohort of veterans.” Here, older cohort could cause some confusion to some readers. When one reads the paper a few years later, he or she probably cannot understand what the older cohort is related to. Authors can add a time frame to it.

Response: This is a thoughtful comment, and we thank the reviewer for these comments and have added age to help support it, page 15, lines 288-290: “Our study found that in a cohort of veterans with an average age of 63 years and a high comorbidity burden, age significantly associated with risk of hospitalization, peak disease severity, and mortality.”

5. Line 131: Similar to point 4, authors should add the Centers for Disease Control and Prevention (CDC) report date.

Response: We have included a date, page 15, lines 291-293: “The CDC provides a list of chronic medical conditions (May 2021) that predispose individuals to severe illness from SARS-CoV-2 infection”

6. Line 133: Authors wrote, “veterans are a unique cohort because of advanced age on average, and more comorbidities. Understanding clinical factors that impact outcomes in veterans will help clinicians risk-stratify patients with similar demographic profiles.” Many veterans could be young in some Veterans Affairs (VA) medical centers. It may be right to general veteran populations, but authors need to cite references to support this claim.

Response: We thank the reviewer for this comment, and we agree that we need to cite a reference for this claim. We have included the following reference, page 16, line 294: Profile of Veterans: 2017. In: National Center for Veterans Analysis and Statistics UDoVA, ed. https://tinyurl.com/2p82akdb

7. Line 137: Authors wrote, “in our study, age was a significant predictor for all of our outcomes and was a confounder for other variables.” Most scientific papers are written from the third point of view. Therefore, it is not common to state the study outcomes as “our outcome.” Authors should use a better phrase, such as in line 151: “This may explain the outcomes in our study.”

Response: We agree with the reviewer and have rephrased this sentence to be, page 16, lines 304 and 305: “In our study, age was a significant predictor for all of the studied outcomes and was a confounder for other variables.”

8. Line 138: Authors wrote, “interestingly, LTC status predicted all three of our outcomes on univariate analysis, but not on multivariate analyses. Earlier in the COVID-19 pandemic, residents of nursing homes had higher rates of infection as well as severe illness and mortality [25].” There is no transition between these two sentences. The first few sentences in the paragraph discussed age as a predictor. However, the sentence “earlier in the COVID-19 pandemic...” did not show an immediate connection with the age issue. Maybe the authors would like to express that nursing homes have older patients. If this is the case, the authors need to provide some connection or background information here.

Response: We do agree that we were trying to say nursing homes may have older patients. We have connected the two ideas, page 16, lines 304-307: “In our study, age was a significant predictor for all of the studied outcomes and was a confounder for other variables. Accordingly, LTC status predicted all three of our outcomes on univariate analysis, but not on multivariate analyses, possibly because LTC units tend to have older residents.”

9. Line 140: Authors wrote that “our study shows that among veterans in LTC facility, disease outcomes were not impacted by their residence status.” Here, authors should provide some discussion or reasons for their findings.

Response: We thank the reviewer for pointing this out. We intended to carry on the previous thought that after adjusting for age, residents of a long-term care (LTC) facility did not have worse outcomes. We have reworded this sentence, page 16, lines 308 and 309: “Our study shows disease outcomes were not impacted by their residence status, after adjusting for age.”

10. Line 148: Authors wrote, “our study supports data from previous reports that non-White patients are at increased risk of hospitalization but have similar peak severity and mortality outcomes [26-29].” Are these non-White patients in the United States or in other countries? This could change the dynamic and purpose of citing the reference. Please clarify.

Response: These studies are from the United States, and we have clarified this point on page 17, lines 315-317: “Our study supports data from previous reports that non-White patients in the United States are at increased risk of hospitalization but have similar peak severity and mortality outcomes.”

11. Line 156: Authors concluded that, for patients with dementia, they could have a high risk of death because of biological factors. Another possibility is the lack of self-report ability in patients with dementia. As a result, they probably do not understand their body’s changes, which could delay the needed care.

Response: We thank the reviewer for this comment and have added in this explanation, page 17, lines 318-321: “This may be explained by a host of biological factors but also may be a result of inability to self-report symptoms. This finding emphasizes the importance of extra care and monitoring required when approaching a patient with dementia.”

12. For the Discussion section, authors may add subtitles to different issues they would like to discuss. The current writing may be a little bit confusing to some readers.

Response: We thank the reviewer for this comment and have added subsections entitled, “Principal Findings,” “Comparison With Previous Studies,” and “Limitations” to our Discussion section.

13. In the Discussion, the authors mentioned multivariate analysis of many potential risk factors as their strength. It is true that the multivariate model is a powerful tool, but it is not necessarily fit for the COVID-19 situation very well. Authors need to cite references about other cases of using the multivariate model for COVID-19 outcome analysis.

Response: We thank the reviewer for this comment and have added several references to other studies using multivariate models after the following sentence in the methods, pages 8 and 9, lines 169-172: “We first conducted a univariate analysis, then used significant variables from the univariate analysis (*P*< 0.05) to use in a multivariate model for each of our outcomes to assess the impact of several variables at once, which has been frequently used in COVID-19 literature.”

Mason KE, Maudsley G, McHale P, Pennington A, Day J, Barr B. Age-Adjusted Associations Between Comorbidity and Outcomes of COVID-19: A Review of the Evidence From the Early Stages of the Pandemic. Front Public Health. 2021;9:584182. PMID: 34422736. doi: 10.3389/fpubh.2021.584182.

Shang W, Dong J, Ren Y, Tian M, Li W, Hu J, et al. The value of clinical parameters in predicting the severity of COVID-19. J Med Virol. 2020 Oct;92(10):2188-92. PMID: 32436996. doi: 10.1002/jmv.26031.

Merzon E, Green I, Shpigelman M, Vinker S, Raz I, Golan-Cohen A, et al. Haemoglobin A1c is a predictor of COVID-19 severity in patients with diabetes. Diabetes Metab Res Rev. 2021;37(5):e3398.

Zhou F, Yu T, Du R, Fan G, Liu Y, Liu Z, et al. Clinical course and risk factors for mortality of adult inpatients with COVID-19 in Wuhan, China: a retrospective cohort study. Lancet. 2020;395(10229):1054-1062.

14. Figures and supplemental tables: Authors should include more details in the titles. Simply writing “genomes” or “hospitalization” in the title is not standard in scientific papers.

Response: We have renamed the titles to be “Hospitalization by patient demographics and comorbidities (%)” and “Mortality by patient demographics and comorbidities (%)”.

15. Figure 1: Authors should provide a better maximum likelihood tree. The current figure has many branches stacked to each other, barely providing any helpful information to readers.

Response: We thank the reviewer for this comment, and we are showing only the branches in which we have a sequence. From this figure, we are hoping to show the diversity of lineages, with the main branch points labeled. For more in-depth information on the exact lineages that our study included, we have provided the frequencies in list format in Multimedia Appendix 1.

## Round 2 Review

### Reviewer W

#### General Comments

The authors of the paper titled “COVID-19 Outcomes and Genomic Characterization of SARS-CoV-2 Isolated From Veterans in New England States: A Retrospective Analysis” have addressed all concerns raised close to full satisfaction. The paper is in much better shape now; however, there still are a few concerns worth noting. Kindly refer to the minor comments.

#### Specific Comments

##### Minor Comments

1. Under “Study Design,” the second and third sentences should be moved to the “Study Setting” and the last sentence moved to “Ethical Considerations.” The justification for the study design initially recommended was to cite any studies on the topic that have used similar methods (if possible).

Response: We have made these changes. We have also cited another study that used similar methods in the veteran population to justify the methodology.

2. Tables 1 and 2 still need to be formatted according to the guidelines.

Response: Tables have been placed where they are referenced in the text and the format is according to instructions. Font size has been changed to normal size, and tables have been reformatted to fit the window in portrait orientation. Soft line breaks have been removed in favor of separate table rows.

3. I still see the captions of figures appearing above the figures, contrary to the guidelines.

Response: We have checked our submitted figures again and did not find captions in the latest submission. All figures are uploaded as supplementary files and follow the journal guidelines. We have removed the older figure files to remove any confusion. The correct files are the supplementary figure files submitted as revised figures on September 14, 2021.

4. Kindly maintain the heading “Multimedia Appendix: Lineages of genomes” in the manuscript but remove the table and upload it in the online manuscript management system.

Response We have removed said table from the manuscript, and it is available as a multimedia appendix in the online manuscript management system. The heading has been maintained in the manuscript as the reviewer suggested.

5. Ensure that all reported percentages in your manuscript are accompanied with the absolute values on which they were calculated, for instance, 25% (5/20) or (25%, 5/20).

Response: Thank you. We have double-checked and added the absolute values where they were missing.

### Reviewer AV

The authors presented an updated manuscript after taking the reviewers’ suggestions. I have a few minor comments.

1. Authors added reference [30] but did not indicate or cite it in the paper. I guess it should be listed here: “which has been frequently used in COVID-19 literature [9,31-33].”

Response: We thank the reviewer for catching this. We have added the citation now.

2. Authors wrote, “this study included all veterans who tested positive for COVID-19 from April 8, 2020, to September 16, 2020 at one of the six New England VA hospitals.” Previously authors wrote, “Connecticut had been entrusted with testing for SARS-CoV-2 for all six VA healthcare centers.” Does this mean the patients enrolled in this study are from one of six VA hospitals, or they are from all six hospitals?

Response: The study included veterans at all six New England VA hospitals. We have clarified this now by changing the word *one* to *any* in the subheading “Participants (Sample Size and Inclusion Criteria).”

3. Authors wrote, “the CDC provides a list of chronic medical conditions (May 2021) that predispose individuals to severe illness from SARS-CoV-2 infection [21], but >75% of United States adults fall under a high-risk category [22].” In general, if the word “but” is in the sentence, readers will pay attention to the words following “but,” which means the first part may not be important or critical. Authors can kindly use another connection word.

Response: We have modified this to the following, to help explain better: “The CDC provides a list of chronic medical conditions (May 2021) that predispose individuals to severe illness from SARS-CoV-2 infection [21]. Based on this list, >75% of United States adults fall under a high-risk category [22], therefore making it important to have select populations evaluated for uniquely applicable risk factors.”

4. In the Abstract, the authors wrote “Multiple SARS-CoV-2 lineages were distributed in patients in New England early in the COVID-19 era, mostly related to viruses from New York with D614G mutation.” Can the authors kindly clarify if it is New York State or New York City?

Response: We have clarified this by adding the word state.

## Abbreviations

AMA: American Medical Association
CDC: Centers for Disease Control and Prevention
LTC: long-term care
ROC: receiver operating characteristic
VA: Veterans Affairs
WGS: whole genome sequencing

## References

[1] Mbwogge M (2021). Peer review of "COVID-19 Outcomes and Genomic Characterization of SARS-CoV-2 Isolated From Veterans in New England States: Retrospective Analysis". JMIRx Med.

[2] de Bruin S, Bos LD, van Roon MA, Tuip-de Boer AM, Schuurman AR, Koel-Simmelinck MJA, Bogaard HJ, Tuinman PR, van Agtmael MA, Hamann J, Teunissen CE, Wiersinga WJ, Koos Zwinderman AH, Brouwer MC, van de Beek D, Vlaar APJ, Amsterdam UMC COVID-19 Biobank Investigators (2021). Clinical features and prognostic factors in Covid-19: a prospective cohort study. EBioMedicine.

[3] Darmon M, Dumas G (2021). Anticipating outcomes for patients with COVID-19 and identifying prognosis patterns. Lancet Infect Dis.

[4] Gutiérrez-Gutiérrez B, Del Toro MD, Borobia AM, Carcas A, Jarrín I, Yllescas M, Ryan P, Pachón J, Carratalà J, Berenguer J, Arribas JR, Rodríguez-Baño J, REIPI-SEIMC COVID-19 group and COVID@HULP groups (2021). Identification and validation of clinical phenotypes with prognostic implications in patients admitted to hospital with COVID-19: a multicentre cohort study. Lancet Infect Dis.

[5] Cummings MJ, Baldwin MR, Abrams D, Jacobson SD, Meyer BJ, Balough EM, Aaron JG, Claassen J, Rabbani LE, Hastie J, Hochman BR, Salazar-Schicchi J, Yip NH, Brodie D, O'Donnell MR (2020). Epidemiology, clinical course, and outcomes of critically ill adults with COVID-19 in New York City: a prospective cohort study. Lancet.

[6] Botta M, Tsonas AM, Pillay J, Boers LS, Algera AG, Bos LDJ, Dongelmans DA, Hollmann MW, Horn J, Vlaar APJ, Schultz MJ, Neto AS, Paulus F, PRoVENT-COVID Collaborative Group (2021). Ventilation management and clinical outcomes in invasively ventilated patients with COVID-19 (PRoVENT-COVID): a national, multicentre, observational cohort study. Lancet Respir Med.

[7] Docherty AB, Harrison EM, Green CA, Hardwick HE, Pius R, Norman L, Holden KA, Read JM, Dondelinger F, Carson G, Merson L, Lee J, Plotkin D, Sigfrid L, Halpin S, Jackson C, Gamble C, Horby PW, Nguyen-Van-Tam JS, Ho A, Russell CD, Dunning J, Openshaw PJ, Baillie JK, Semple MG, ISARIC4C investigators (2020). Features of 20 133 UK patients in hospital with covid-19 using the ISARIC WHO Clinical Characterisation Protocol: prospective observational cohort study. BMJ.

[8] Bastard P, Rosen LB, Zhang Q, Michailidis E, Hoffmann HH, Zhang Y, Dorgham K, Philippot Q, Rosain J, Béziat V, Manry J, Shaw E, Haljasmägi L, Peterson P, Lorenzo L, Bizien L, Trouillet-Assant S, Dobbs K, de Jesus AA, Belot A, Kallaste A, Catherinot E, Tandjaoui-Lambiotte Y, Le Pen J, Kerner G, Bigio B, Seeleuthner Y, Yang R, Bolze A, Spaan AN, Delmonte OM, Abers MS, Aiuti A, Casari G, Lampasona V, Piemonti L, Ciceri F, Bilguvar K, Lifton RP, Vasse M, Smadja DM, Migaud M, Hadjadj J, Terrier B, Duffy D, Quintana-Murci L, van de Beek D, Roussel L, Vinh DC, Tangye SG, Haerynck F, Dalmau D, Martinez-Picado J, Brodin P, Nussenzweig MC, Boisson-Dupuis S, Rodríguez-Gallego C, Vogt G, Mogensen TH, Oler AJ, Gu J, Burbelo PD, Cohen JI, Biondi A, Bettini LR, D'Angio M, Bonfanti P, Rossignol P, Mayaux J, Rieux-Laucat F, Husebye ES, Fusco F, Ursini MV, Imberti L, Sottini A, Paghera S, Quiros-Roldan E, Rossi C, Castagnoli R, Montagna D, Licari A, Marseglia GL, Duval X, Ghosn J, Tsang JS, Goldbach-Mansky R, Kisand K, Lionakis MS, Puel A, Zhang SY, Holland SM, Gorochov G, Jouanguy E, Rice CM, Cobat A, Notarangelo LD, Abel L, Su HC, Casanova JL, HGID Lab, NIAID-USUHS Immune Response to COVID Group, COVID Clinicians, COVID-STORM Clinicians, Imagine COVID Group, French COVID Cohort Study Group, Milieu Intérieur Consortium, CoV-Contact Cohort, Amsterdam UMC Covid-19 Biobank, COVID Human Genetic Effort (2020). Autoantibodies against type I IFNs in patients with life-threatening COVID-19. Science.

[9] Zhou F, Yu T, Du R, Fan G, Liu Y, Liu Z, Xiang J, Wang Y, Song B, Gu X, Guan L, Wei Y, Li H, Wu X, Xu J, Tu S, Zhang Y, Chen H, Cao B (2020). Clinical course and risk factors for mortality of adult inpatients with COVID-19 in Wuhan, China: a retrospective cohort study. Lancet.

[10] Lee M, Sallah YH, Petrone M, Ringer M, Cosentino D, Vogels CBF, Fauver JR, Alpert TR, Grubaugh ND, Gupta S (2021). COVID-19 outcomes and genomic characterization of SARS-CoV-2 isolated from veterans in New England states: retrospective analysis. JMIRx Med.

[11] Rodriguez-Morales AJ, Balbin-Ramon GJ, Rabaan AA, Sah R, Dhama K, Paniz-Mondolfi A, Pagliano P, Esposito S (2020). Genomic Epidemiology and its importance in the study of the COVID-19 pandemic. Infez Med.

[12] Fehr AR, Perlman S (2015). Coronaviruses: an overview of their replication and pathogenesis. Methods Mol Biol.

[13] The Lancet (2021). Genomic sequencing in pandemics. Lancet.

[14] What are JMIR's guidelines for article titles?. JMIR Publications Knowledge Base and Help Center.

[15] Instructions for authors of JMIR. JMIR Publications.

[16] (2021). Genomic sequencing of SARS-CoV-2: a guide to implementation for maximum impact on public health. World Health Organization.

[17] Field N, Cohen T, Struelens MJ, Palm D, Cookson B, Glynn JR, Gallo V, Ramsay M, Sonnenberg P, Maccannell D, Charlett A, Egger M, Green J, Vineis P, Abubakar I (2014). Strengthening the Reporting of Molecular Epidemiology for Infectious Diseases (STROME-ID): an extension of the STROBE statement. Lancet Infect Dis.

[18] Benchimol EI, Smeeth L, Guttmann A, Harron K, Moher D, Petersen I, Sørensen HT, von Elm E, Langan SM, RECORD Working Committee (2015). The REporting of studies Conducted using Observational Routinely-collected health Data (RECORD) statement. PLoS Med.

[19] How should tables be formatted?. JMIR Publications Knowledge Base and Help Center.

[20] What are the guidelines for supplementary files (figures, multimedia appendices, additional material for reviewers/editors only)?. JMIR Publications Knowledge Base and Help Center.

[21] (2021). People with certain medical conditions. Centers for Disease Control and Prevention.

[22] Ajufo E, Rao S, Navar AM, Pandey A, Ayers CR, Khera A (2021). U.S. population at increased risk of severe illness from COVID-19. Am J Prev Cardiol.

[23] (2017). Profile of veterans: 2017. United States Department of Veterans Affairs.

[24] Guo L (2021). Peer review of "COVID-19 Outcomes and Genomic Characterization of SARS-CoV-2 Isolated From Veterans in New England States: Retrospective Analysis". JMIRx Med.

[25] Bagchi SMJ, Mak J, Li Q, Sheriff E, Mungai E, Anttila A, Soe MM, Edwards JR, Benin AL, Pollock DA, Shulman E, Ling S, Moody-Williams J, Fleisher LA, Srinivasan A, Bell JM (2021). Rates of COVID-19 among residents and staff members in nursing homes - United States, May 25-November 22, 2020. MMWR Morb Mortal Wkly Rep.

[26] Cardemil CV, Dahl R, Prill MM, Cates J, Brown S, Perea A, Marconi V, Bell L, Rodriguez-Barradas MC, Rivera-Dominguez G, Beenhouwer D, Poteshkina A, Holodniy M, Lucero-Obusan C, Balachandran N, Hall AJ, Kim L, Langley G (2021). COVID-19-related hospitalization rates and severe outcomes among veterans from 5 Veterans Affairs medical centers: hospital-based surveillance study. JMIR Public Health Surveill.

[27] Gold JAW, Wong KK, Szablewski CM, Patel PR, Rossow J, da Silva J, Natarajan P, Morris SB, Fanfair RN, Rogers-Brown J, Bruce BB, Browning SD, Hernandez-Romieu AC, Furukawa NW, Kang M, Evans ME, Oosmanally N, Tobin-D'Angelo M, Drenzek C, Murphy DJ, Hollberg J, Blum JM, Jansen R, Wright DW, Sewell WM, Owens JD, Lefkove B, Brown FW, Burton DC, Uyeki TM, Bialek SR, Jackson BR (2020). Characteristics and clinical outcomes of adult patients hospitalized with COVID-19 - Georgia, March 2020. MMWR Morb Mortal Wkly Rep.

[28] Kabarriti R, Brodin NP, Maron MI, Guha C, Kalnicki S, Garg MK, Racine AD (2020). Association of race and ethnicity with comorbidities and survival among patients with COVID-19 at an urban medical center in New York. JAMA Netw Open.

[29] Azar KMJ, Shen Z, Romanelli RJ, Lockhart SH, Smits K, Robinson S, Brown S, Pressman AR (2020). Disparities in outcomes among COVID-19 patients in a large health care system in California. Health Aff (Millwood).

[30] Mason KE, Maudsley G, McHale P, Pennington A, Day J, Barr B (2021). Age-adjusted associations between comorbidity and outcomes of COVID-19: a review of the evidence from the early stages of the pandemic. Front Public Health.

[31] Al-Salameh A, Lanoix JP, Bennis Y, Andrejak C, Brochot E, Deschasse G, Dupont H, Goeb V, Jaureguy M, Lion S, Maizel J, Moyet J, Vaysse B, Desailloud R, Ganry O, Schmit JL, Lalau JD (2021). Characteristics and outcomes of COVID-19 in hospitalized patients with and without diabetes. Diabetes Metab Res Rev.

[32] Shang W, Dong J, Ren Y, Tian M, Li W, Hu J, Li Y (2020). The value of clinical parameters in predicting the severity of COVID-19. J Med Virol.

[33] Merzon E, Green I, Shpigelman M, Vinker S, Raz I, Golan-Cohen A, Eldor R (2021). Haemoglobin A1c is a predictor of COVID-19 severity in patients with diabetes. Diabetes Metab Res Rev.

